# Nutritional and Microbiome Effects of a Partial Substitution of Poultry Meat with Hydrolyzed Feather Meal in Dog Diets

**DOI:** 10.3390/microorganisms13010121

**Published:** 2025-01-09

**Authors:** Fatemeh Balouei, Bruno Stefanon, Rosangela Armone, Andrea Randazzo, Biagina Chiofalo

**Affiliations:** 1 Department of Agrifood, Environmental and Animal Science, University of Udine, Via delle Scienze 206, 33100 Udine, Italy; balouei.fatemeh@spes.uniud.it; 2 Department of Veterinary Sciences, University of Messina, Via Palatucci Snc, 98168 Messina, Italy; rosangela.armone@studenti.unime.it (R.A.); a_randazzo76@yahoo.it (A.R.); biagina.chiofalo@unime.it (B.C.)

**Keywords:** hydrolyzed feather meal, fecal microbiota, dog, sex

## Abstract

Two extruded diets isoenergetic, isonitrogenous, and isolipidic were formulated with poultry meal (control diet) as the source of animal-origin proteins (160 g/kg of feed) or with 90 g/kg of poultry meal and 70 g/kg of hydrolyzed feather meal (treated diet) and were fed to eight dogs (four adult female and four adult male English Setters). Body condition, muscle condition, and fecal consistency scores and body weight were monitored at the beginning of the trial and after 3, 7, 15, and 45 days, and no significant differences (*p* > 0.05) were observed between diets and between sex. Fecal samples, collected at the same time points, were analyzed for microbiota composition. No significant difference was calculated for the alpha diversity index between control diet and treated diet, nor for the diets × times of sampling interaction and for sex. Beta diversity was different (*p*-value 0.001) between the control and treated groups. The beta diversity between sexes was significantly different (*p*-value = 0.047). Linear Discriminant analysis effect size analysis revealed significant differences between dietary groups, identifying *Clostridiales*, *Coprococcus*, *Bacteroides plebeius*, *Eubacterium biforme*, *Catenibacterium*, and *Prevotella copri* as more abundant in the CTR diet, while *Fusobacterium, Bacteroides, Fusobacteriaceae*, *Paraprevotellaceae, Enterococcus*, *Faecalibacterium*, *Enterobacteriaceae*, *Peptostreptococcaceae*, and *Clostridium spiroforme* were more abundant in the treated diet. Sex differences were also significant, with 25 taxa differing between male and female dogs. Overall, the study underscores the impact of HFM and sex on gut microbiota composition in dogs, with potential implications for dietary interventions and microbiome research.

## Simple Summary

This study investigated the use of hydrolyzed feather meal as a substitute for poultry meat in dog diets. Two groups of four English Setters, two male and two female each, were fed two different diets over 45 days. The control group was fed with poultry meal as the protein source and one treated group with a mix of poultry meal and hydrolyzed feather meal. Body weight, body condition, muscle condition, and fecal consistency scores did not differ between groups and sex. Beta diversity varied significantly between the control and treated groups, as well as between sex. Fourteen bacterial taxa were different between diet and 25 between sex. Overall, the study highlights the influence of hydrolyzed feather meal and sex on gut microbiota in dogs, suggesting potential implications for dog nutrition and microbiome research.

## 1. Introduction

The microbiota performs various biochemical functions that influence the host, such as producing metabolites, regulating physiological and biochemical processes, and modulating the immune system [1]. The constantly changing gastrointestinal environment greatly affects the composition, function, and metabolism of the intestinal microbiota [2]. The microorganisms found in the gastrointestinal tract of domestic dogs form a diverse and intricate community [3,4]. The commensal bacteria found in a dog’s gastrointestinal tract belongs to one of the five predominant bacterial phyla, *Bacteroidetes*, *Fusobacteria*, *Firmicutes*, *Proteobacteria*, and *Actinobacteria* [5]. Generally, these communities reflect the environment, substrate availability and functions of the specific region of the gastrointestinal tract they occupy [6,7,8].

Diet is the most crucial factor shaping the intestinal environment, making the microbiota highly sensitive to dietary changes (Lin et al., 2022) [9]. The microbiota aids in food breakdown, fermentation of complex carbohydrates and amino acids, with the production of short-chain fatty acids (SCFAs) [10]. The food consumed by the host is the primary nourishment for the intestinal microbiota, significantly shaping its composition. This microbiota, in turn, influences not only the host’s gut function (Rhimi et al., 2022; Soontararak et al., 2019) [11,12] but also regulate the immune and the neuroendocrine responses and, more generally, the health of animal (Jefferey et al., 2017; Kiełbik et al., 2024; Montserrat-Malagarriga et al., 2024) [13,14,15]. Both humans and animals need a balanced intake of carbohydrates, proteins, fats, and metabolites for health. Excess or indigestible food bypasses initial digestion and is broken down in the colon by intestinal microbes [16]. In dogs obligate anaerobic bacteria, for instance, are primarily located in the large anaerobic intestine and mainly belong to the *Firmicutes* phylum and/or have ability to ferment dietary fibers [6,8,17]. In comparison, the oxygen-rich small intestine hosts aerobic and facultative anaerobic bacteria, along with protein-metabolizing bacteria, which typically belong to the *Proteobacteria* phylum [3,6,8]. Generally, a high prevalence of *Fusobacteria* is linked to protein-rich diets [18,19,20], while an increased relative abundance of *Proteobacteria* is associated with protein metabolism in dogs [3].

Considering the importance of diet on the microbial community of the gut in dogs [5,21], the effect of nutrients or ingredients composition of the diet can be evaluated also through the analysis of fecal microbiota. This technique offers the opportunity to carry out dietary intervention studies without the need to handle animals, since they require only fecal collection from the floor. Furthermore, the analysis of fecal quality and microbiota can be extended to assess the inclusion of byproducts of agrifood industry into the diet (Cabrita et al., 2024; Chuppava et al., 2023; Guilherme-Fernandes et al., 2024) [22,23,24], that is paramount for the evaluation of circular economy. This approach was recently applied to investigate the effect of hydrolyzed feather meal as a protein source in dogs [25].

The quantities of animal parts in category 3 in the EU (Regulation (EC) 1069/2009 49) [26] amount to around 18 million tonnes per year (Chuppava et al., 2023) [23]. Animal byproducts are used as raw materials for pet food and come mainly from the slaughter of cattle, pigs, and chickens and rom fish industry. These ingredients are essential for the pet food industry and provide most of the protein included in diets, with benefits to the environment and a circular economy perspective (Vanelli et al., 2021) [27]. It is therefore very important to study the nutritional value of byproducts in order to assess the effect of their inclusion on digestive behaviour and intestinal health.

Treated feather and hog hair meals are valuable protein sources for growing chicks [28]. Enzyme supplementation, including protease and amylase, improves poultry growth performance [29]. Keratinase, an enzyme that breaks down various proteins [30], aids in the degradation of chicken feathers and keratinous waste by fungi [31], which can then be used as nitrogen-rich fertilizers [32,33]. This microbial degradation provides an alternative protein source, reduces production costs, and converts feather waste into a digestible feather meal [34].

This study aimed to assess the impact of incorporating hydrolyzed feather meals (HFM) as a substitute for poultry meat on the fecal microbiome of dogs, using 16S rRNA ribosomial amplicon sequencing to analyze microbial diversity and abundance.

## 2. Materials and Methods

### 2.1. Animal Ethics Statement

The research was approved by the Ethical Animal Care and Use Committee of the University of Messina, Department of Veterinary Sciences (24 January 2023, code 01/2023), and the experiment was carried out following ethical and animal welfare guidelines.

### 2.2. Animals and Diets

Two extruded diets, isoenergetic (calculated metabolizable energy of 3680 kcal/kg, as-fed), isonitrogenous (19%, as-fed) and isolipidic (15%, as-fed) were formulated to meet the nutritional requirements of adult dogs [35]. One diet, CTR, was formulated with poultry meal (PM) as the source of proteins of animal origin (16% of feed) and one diet, TRT, was formulated with 9% poultry meal (PM) and 7% of HFM with pressure and steam (GOLDMEHL FM, Gepro, Diepholz, Germany). GOLDMEHL FM contains approximately 8.3% crude protein, 0.7% crude fat, 0.1% of crude fiber, 0.6% starch, and 0.2% ash (as-fed) and a declared ileal digestible protein > 80%. GOLDMEHL FM originates from healthy poultry byproducts, and it is considered a processed animal protein, Cat. III material (Regulation EC No 1069/2009) [26].

The amount of calcium and phosphorus of the diet were adjusted, due to the lower quantity in HFM compared to PM. Diets also contained docosahexaenoic acid (DHA). The formulation and the chemical composition of diets is reported in Table 1 [25]. The amino acids content of the diets is also shown in Table 2.

The research was carried out with four adult female English setter dogs, divided into two groups, TRT group and CTR group, with comparable initial body weight (BW, TRT: 16.8 kg; CTR: 16.1 kg), body condition score (BCS: 5, on a nine-point scale), muscle condition score (MCS: 1, on a four-point scale) and fecal consistency score (FCS: 2.25, on a five-point scale), and four adult male English setter dogs, divided into two groups: TRT group and CTR group with comparable initial body weight (TRT: 21.6 kg; CTR: 21.9 kg), BCS (5, on a nine-point scale), MCS (1, on a four-point scale) and FCS (2.3, on a five-point scale).

Body weight was recorded at 9:00 a.m. from fasted animals with a platform electronic balance (EOS 150K100NXL, Kern and Sohn GMBH; Balingen—Germany). The BCS was attributed utilizing a rating scale from one (too thin) to nine (too heavy), according to the table of the WSAWA Global Nutrition Guidelines [36]. The MCS was assessed with a scale ranging from one (no muscle wasting and normal muscle mass) to four (marked muscle wasting), according to the table of the WSAWA Global Nutrition Guidelines. Evaluation of muscle mass encompassed visual examination and palpation of scapulae, ribs, lumbar vertebrae, temporal, and pelvic bones [37]. Fecal consistency score was subjectively evaluated using a rating scale ranging from one (dry stool) to five (liquid stool), based on the Waltham Fecal Score table [38].

Dogs, privately owned, were kept in the same environmental conditions. The dogs were housed in cages with natural light–dark cycle and allowed to exercise in an outdoor area in the morning and evening, for about 1 h each time. All the dogs had regular opportunities for socializing with each other and with members of the owner’s family. A clinical assessment including physical examinations, complete blood counts, biochemical tests, and fecal analyses, was performed for each dog before the beginning of the study, to assess their health conditions. Dogs underwent diagnosis of *Leishmania infantum* using the Indirect Immune Fluorescent Antibody Test (IFAT). A concomitant coprological analysis was carried out to detect the presence of endoparasites. The study lasted 45 days (from 9 October to 24 November), with 7 days for adaptation to the diets (T-15) as recommended by the American Animal Hospital Association [39].

The daily diet fed to each dog was calculated based on the calculated metabolizable energy requirements for adult dogs with moderate physical activity (125 × BW ^0.75^ kcal for 1–3 h/day), as suggested by the European Pet Food Industry Federation guidelines [35] in relation to the caloric density of metabolizable energy (ME) of each diet (CTR and TRT).

The amounts of diets were administered individually once a day, at 8.00 p.m., according to the FEDIAF guidelines [35].

The protocol of the study was carefully controlled, considering the factors for both groups, ensuring a comprehensive and controlled approach.

### 2.3. Data Recording and Samples Collections

Over the 45-day period, day 0 (T0), day 3 (T03), day 7 (T07), day 15 (T15), day 45 (T45), for each dog the BW, BCS, and MCS were recorded. For microbiota sequencing, fecal samples were collected on the following pairs of days: day 0 and day 1 for T0, day 3 and day 4 for T03, day 7 and day 8 for T07, day 15 and day 16 for T15, and day 45 and day 46 for T45. Screw cap tubes containing eNAT^®^ transport and storage medium (eNAT^®^ tubes, Copan, Brescia, Italy) were used to collect fecal samples at 9:00 a.m. on consecutive days. They were promptly stored in the dark at room temperature awaiting analysis, adhering to the preservation requirements of the eNAT^®^ medium.

### 2.4. Microbiota Analysis

DNA extraction was performed within 3 weeks from the collection date. Total DNA extraction for microbiome analysis was performed on 150 mg of feces using the Quick-DNA™ Fecal/Soil Microbe Miniprep Kit (Zymo Research, Irvine, CA, USA), following the manufacturer’s instructions. Quantification and quality check of the DNA were carried out using a QubitTM 3 Fluorometer (Thermo Scientific; Waltham, MA, USA). Following DNA extraction, libraries were prepared by amplifying the hypervariable regions V3 and V4 of the 16S rRNA (primers: 341F CCTAYGGGRBGCASCAG and 806R GGACTACNNGGGTATCTAAT) and incorporating sequencing indexes. This step utilized the NEBNext^®^ Ultra™ IIDNA Library Prep Kit (Cat No. E7645), following the manufacturer’s guidelines. The resulting amplicons underwent sequencing on a Novaseq 6000 platform, SP flow cell (Illumina; San Diego, CA, USA) in 2 × 250 paired-end mode, following standard protocols for an intended depth of sequencing of 50,000 reads per sample. The raw sequence data obtained was deposited in the NCBI Sequence Read Archive under the accession number PRJNA1079213.

### 2.5. Bioinformatic

The raw sequences (FASTQ) of the samples were processed using the bioinformatics tool Quantitative Insights into Microbial Ecology 2 (QIIME 2) [40]. The following steps were undertaken: demultiplexing was performed, sequenced reads meeting the quality threshold (Phred score ≥ 30) were identified, denoising with DADA2, and chimeras were filtered out. Overall, a total of 4,449,601 reads with an average count per sample of 55,620 reads and lowest count of 32,450 reads. These sequences of high-quality were clustered into amplicon sequence variants (ASVs) and annotated against the greengenes database [41] for 16S rRNA.

### 2.6. Statistical Analysis

Reads were assigned to taxa until specie level and the data were uploaded to the Microbiome Analyst (https://www.microbiomeanalyst.ca/ (accessed on 12 October 2024) for statistical and integrative analysis of microbiome [42] and normalized as (RA), based on rarefaction to the minimum read counts of samples. The reads RA were used to compute Shannon alpha diversity and the comparisons for the factors Diet, Diet X Times of sampling, and Sex were tested with Mann–Whitney test, with multi-testing adjustment based on Benjamini–Hochberg procedure (FDR). Bray–Curtis beta diversity differences between Diet, Diet X Times of sampling, and Sex were also computed, and the results were visualized using Principal Coordinate Analysis (PCoA) plots. Permutational multivariate analysis of variance (PERMANOVA) was applied to evaluate differences in community composition. Linear Discriminant Analysis Effect Size (LEfSe) was subsequently applied [43] to compare RA between Diets, Diets X Times of sampling, and Sex.

Data of BCS, MCS, and FCS were analyzed with ordinal regression models, with the effects of diet, sex, time of sampling, and interaction of diet X time of sampling. Data of BW were analyzed with mixed model, with the fixed effects of diet, sex, and time of sampling and the interaction of diet X time of sampling and with the random effect of dog. These statistical analyses were performed with XLSTAT (Addinsoft, Paris, France, 2022).

## 3. Results

### 3.1. Animal Performances

In relation to the diet, the BW, BCS, and MCS did not significantly vary during the study between the dogs fed the CTR and TRT diets (Table 3). The TRT diet caused a slight but not significant (*p* > 0.05) decrease in FCS compared to the CTR diet. In relation to the sex, the BW of males was significantly higher (*p* < 0.01) than females, as expected, while no significant differences were observed for BCS, MCS, and FCS. The interaction diet x time of sampling showed significantly (*p* < 0.05) lower values for the FCS in the TRT group at T0, T7, and T15 compared to T3 and T45, and also lower than the values measured for the CTR group. However, at T45, the FCS of the CTR and TRT groups did not significantly differ. Overall, the mean values of BCS, MCS, and FCS indicated generally good health conditions.

### 3.2. Microbiome

#### 3.2.1. Relative Abundances

**Figure 1:**
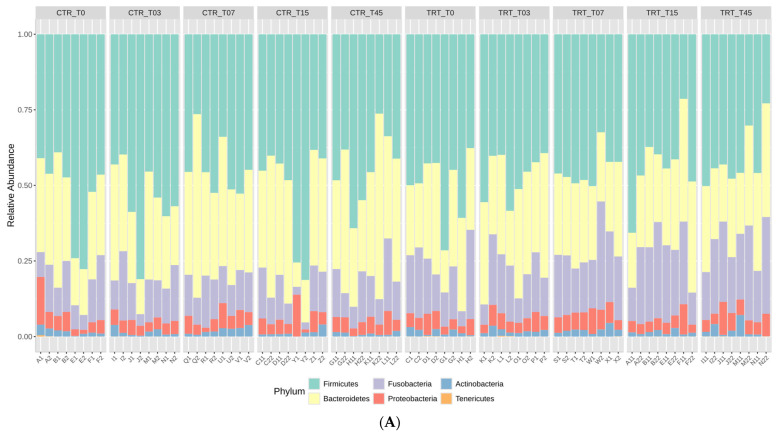

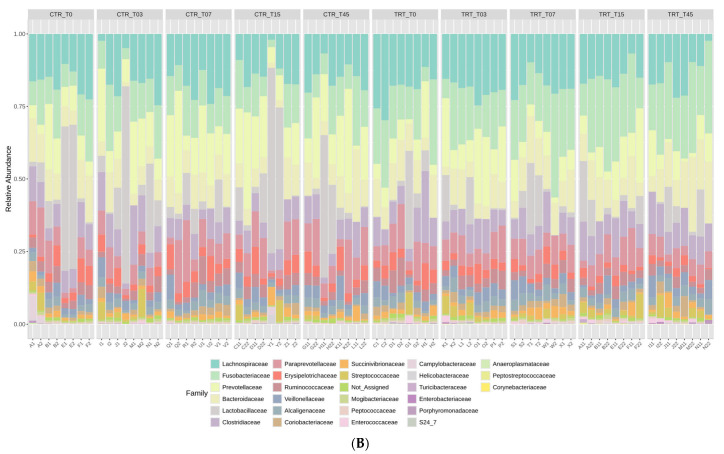
Relative abundance profile of phyla (Panel **A**) and families (Panel **B**) of the dogs fed with control diet (CTR) and experimental diet (TRT) at T0, T03, T07, T15, and T45. CTR group, dogs fed with diet with poultry meal; TRT group, dogs fed with diet with hydrolyzed feather meal. Sampling times: T0, T03, T07, T15, and T45 indicate samples collected at the beginning of the study and after 3, 7, 15, and 45 days.

The rarefaction curves, reported in the Supplementary Material Figure S1, showed that the sequence depth was satisfactory for all the samples in the CTR and TRT diets. The results of relative abundance (RA) at the phylum and family taxonomic levels are reported in Figure 1 for the two diets at the time points. These results indicated that the phyla *Firmicutes*, *Bacteroidetes*, and *Fusobacteria* were the most represented in the feces of the dogs (Figure 1A). At the family taxonomic level, *Lachnospiraceae*, *Fusobacteriaceae*, *Prevotellaceae*, and *Bacteroidaceae* were the most dominant families in the fecal microbiota of dogs (Figure 1B).

#### 3.2.2. Alpha Diversity

**Figure 2:**
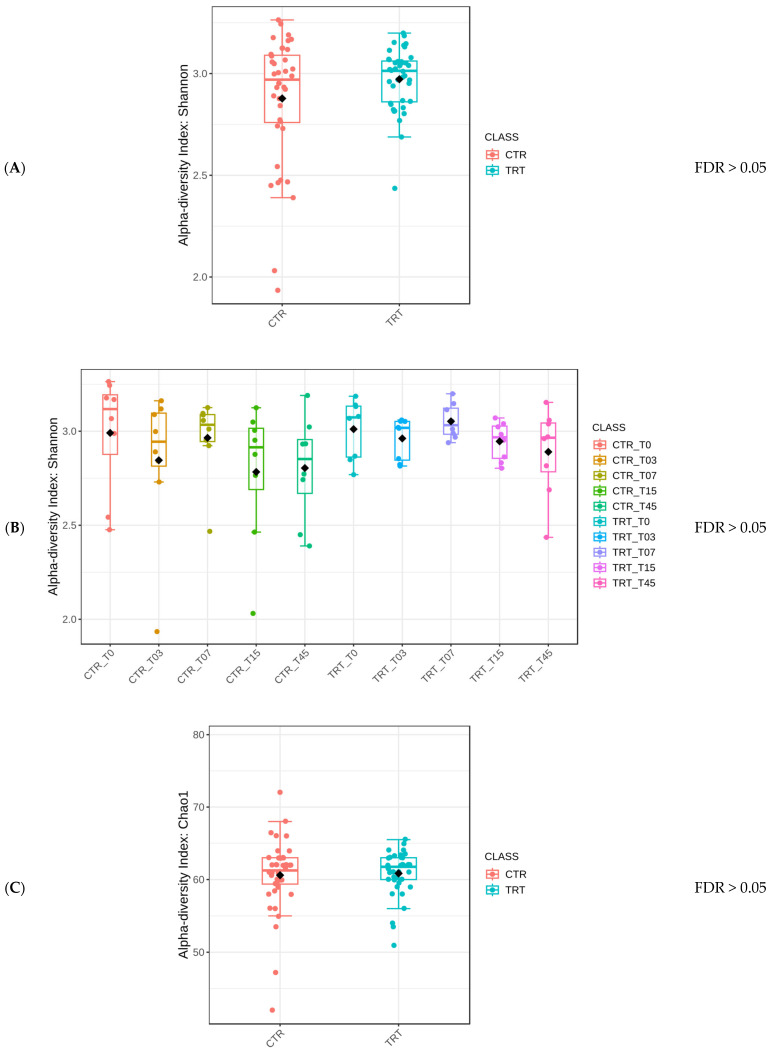
Comparison of the Shannon alpha diversity index of dogs across dietary treatments (Panel **A**), Diet × Time of sampling interaction (Panel **B**), and Sex (Panel **C**). CTR group, dogs fed with diet with poultry meal; TRT group, dogs fed with diet with hydrolyzed feather meal. Sampling times: T0, T03, T07, T15, and T45 indicate samples collected at the beginning of the study and after 3, 7, 15, and 45 days.

The Mann–Whitney test yielded an FDR > 0.05, indicating no statistically significant difference in the Shannon alpha diversity index between the two dietary groups, marked as CTR and TRT (Figure 2A). Similarly, there was no statistically significant difference for the Diet × Time of sampling interaction (Figure 2B) or for Sex (Figure 2C).

#### 3.2.3. Beta Diversity

**Figure 3:**
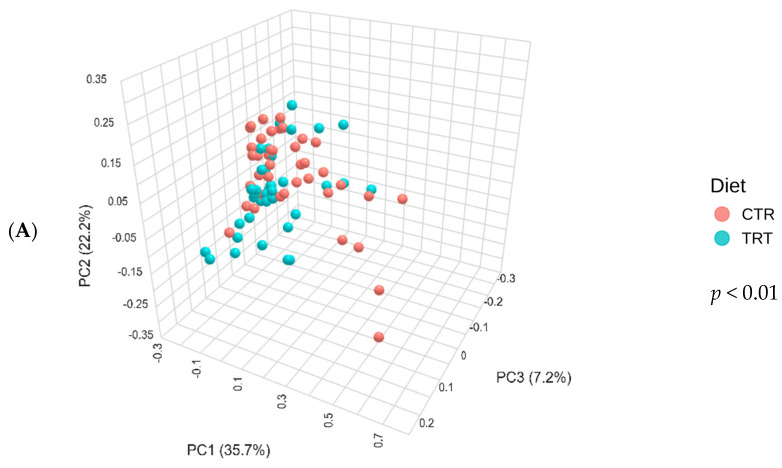

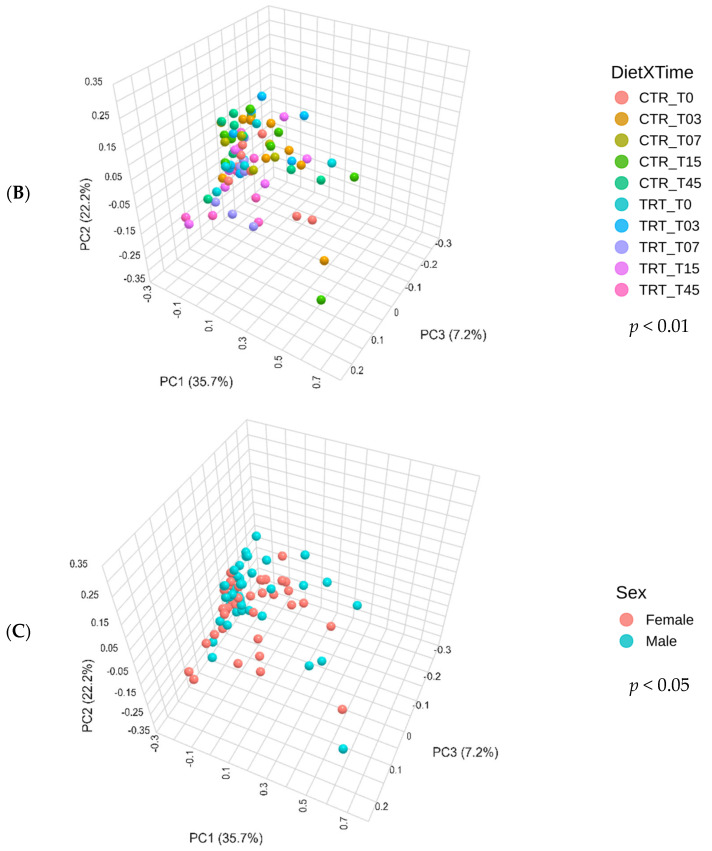
Principal component analysis of the Bray–Curtis beta diversity index of dogs across dietary treatments (Panel **A**), Diet × Time of sampling interaction (Panel **B**), and Sex (Panel **C**). CTR group, dogs fed with diet with poultry meal; TRT group, dogs fed with diet with hydrolyzed feather meal. Sampling times: T0, T03, T07, T15, and T45 indicate samples collected at the beginning of the study and after 3, 7, 15, and 45 days.

The CTR group, consisting of dogs fed the PM diet (diet with poultry meal), and the TRT group, consisting of dogs fed the HFM diet (diet with hydrolyzed feather meal), were analyzed for differences in beta diversity using the Bray–Curtis dissimilarity matrix, reported as PCoA (Figure 3A). The PERMANOVA test yielded a *p*-value of 0.001, indicating a statistically significant difference in the Bray–Curtis beta diversity index between the two dietary groups.

Figure 3B shows the Bray–Curtis beta diversity index for dogs fed with CTR and TRT diets throughout the study period. The CTR and TRT groups were examined at several time points: T0, T03, T07, T15, and T45. The PERMANOVA test found a *p*-value of 0.009, which means there were significant differences in beta diversity between the two diets over the study period. However, significant differences were only detected in pairwise comparisons between CTR_T07 and TRT_T07 and between CTR_T07 and TRT_T45, showing differences in beta diversity at these specific times.

Figure 3C shows the beta diversity index for male and female dogs. The PERMANOVA test revealed a significant difference in the Bray–Curtis beta diversity index between sexes, with a *p*-value of 0.047.

#### 3.2.4. LEfSe Analysis

**Figure 4:**
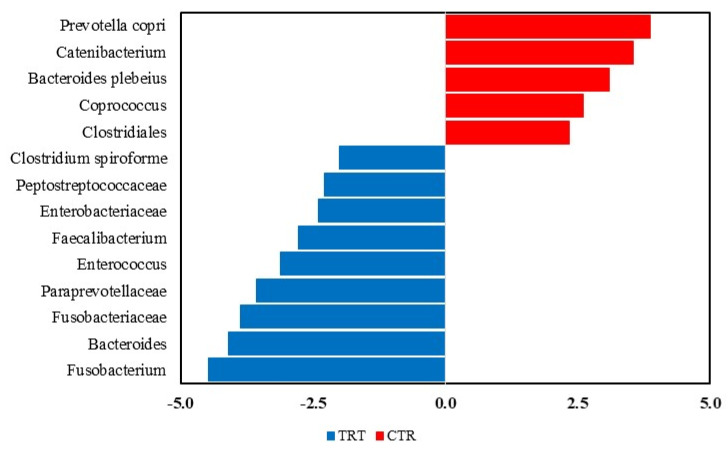
Taxa that significantly differed between the CTR and TRT diets in the Linear Discriminant Analysis Effect Size (LEfSe). CTR group, dogs fed with diet with poultry meal; TRT group, dogs fed with diet with hydrolyzed feather meal.

**Figure 5:**
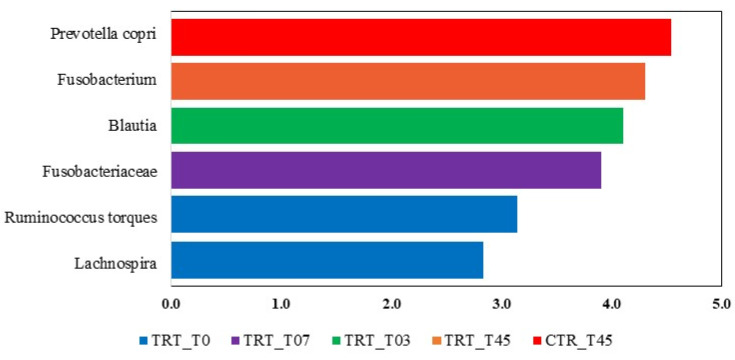
Taxa that significantly differed in the Linear Discriminant Analysis Effect Size (LEfSe) between the CTR and TRT diets during the sampling times. CTR group, dogs fed with diet with poultry meal; TRT group, dogs fed with diet with hydrolyzed feather meal. Sampling times: T0, T03, T07, T15, and T45 denote samples collected at the beginning of the study and after 3, 7, 15, and 45 days.

The LEfSe analysis revealed significant differences in the fecal microbiota composition of dogs fed with the CTR and TRT diets group (Figure 4). In the CTR diet group, the order Clostridiales and four genera were more abundant. In contrast, in the TRT diet group, four families, *Fusobacteriaceae*, *Paraprevotellaceae*, *Enterobacteriaceae*, *Peptostreptococcaceae*, and seven genera were prevalent. Based on the LEfSe analysis of the significant features identified in the Diet x Time interaction (Figure 5), it was shown that on day 45, dogs fed the CTR diet group exhibited a significant abundance of the species *Prevotella copri*. At T0, dogs fed the TRT diet group had a significant abundance of the genus Fusobacterium. For the TRT diet group, the genus *Blautia* showed a significant increase in abundance at T03, and the family *Fusobacteriaceae* did so at T07. Furthermore, the LDA scores of the genera were above 4.

**Figure 6:**
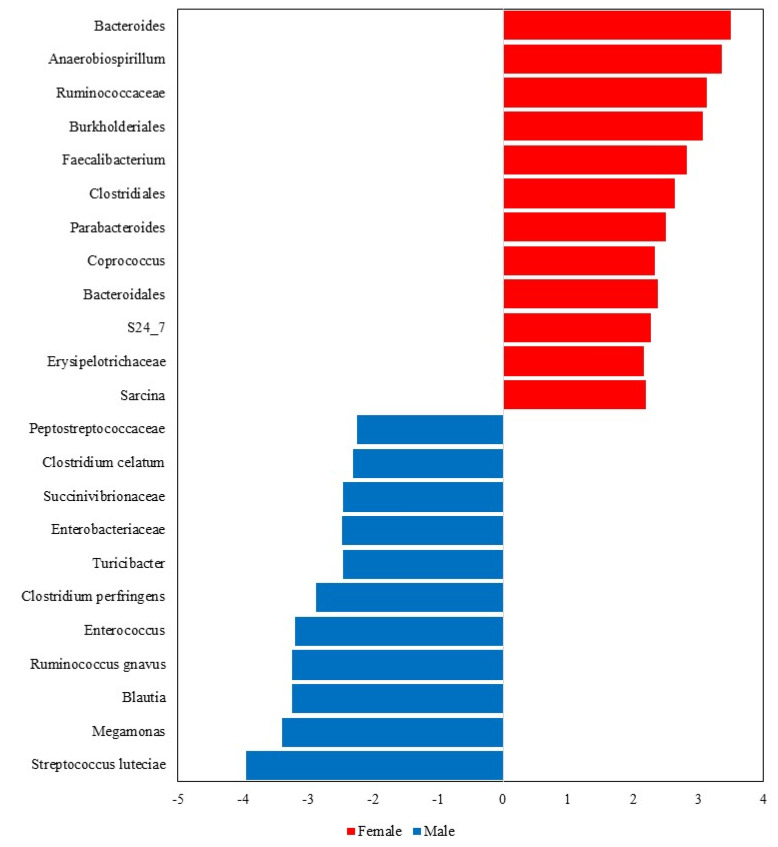
Taxa that significantly differed in the Linear Discriminant Analysis Effect Size (LEfSe) between male and female dogs.

The LEfSe analysis further highlighted notable differences in the fecal microbiota composition between male and female dogs (Figure 6). Overall, 23 taxa were significantly different between sexes, with 12 taxa being more abundant in females (*Bacteroides*, *Anaerobiospirillum*, *Ruminococcaceae*, *Burkholderiales*, *Faecalibacterium, Clostridiales*, *Parabacteroides*, *Coprococcus*, *Bacteroidales*, S24_7, *Erysipelotrichaceae and Sarcina*) and 11 in males (*Peptostreptococcaceae*, *Clostridium celatum, Succinivibrionaceae*, *Enterobacteriaceae*, *Turicibacter, Clostridium perfringens, Enterococcus, Ruminococcus gnavus, Blautia, Megamonas,* and *Streptococcus luteciae*).

## 4. Discussion

### 4.1. Animal Performances

The results of BW, BCS, and MCS, which did not vary between TRT and CTR diets, align with a previous publication by Balouei et al. [25] and another study by Wahab et al. [44], which investigated the addition of 5%, 10%, and 20% HFM in the diet fed to adult female Beagles. In relation to sex, Wallis et al. [45] hypothesized that a sex-specific effect on BCS exists in canine species, being a notable factor for canine obesity. They demonstrated how this might occur: in their study, female Labrador retrievers had a higher mean BCS than male Labrador retrievers. Importantly, this sex effect strongly depended on whether the dogs were intact (female BCS > male BCS) or neutered (male BCS > female BCS).

The significant differences in FCS between groups (*p* < 0.05) at some times of sampling are partly in agreement with the previous studies by Balouei et al. [25] and Pacheco et al. [46], which involved adult Beagles fed diets with 7.5% and 15% HFM. Indeed, El- Wahab et al. [44] reported an increase in FCS with inclusion levels of 10% and 20% HFM in the diet of Beagle dogs. Deschamps et al. [47] noted that dog size influences digestion and microbiota, which may partly explain the differences between the present study and those conducted with Beagles. However, other factors, such as age, sex, previous dietary regimes, and environmental conditions, could also be directly or indirectly involved. Nevertheless, the numerical differences in FCS values between the two groups were limited to well-formed feces for all dogs over time (T0 to T45) with fecal scores ranging from 2.2 to 2.5 on a five-point scale testifying normal gut health.

### 4.2. Fecal Microbiome

The impact of substituting PM with HFM in the diets of adult male and female dogs on their gut microbiome was assessed at various time points during the trial. You and Kim [48] found that healthy dogs predominantly harbor *Firmicutes*, *Bacteroidetes*, *Fusobacteria*, *Proteobacteria*, and *Actinobacteria*, with *Fusobacterium* abundance varying by breed and age. Studies using next-generation sequencing [3] reveal that healthy dogs and cats have gut microbiomes rich in *Firmicutes* and *Bacteroidetes*, with a notable presence of *Proteobacteria*, which includes both beneficial and opportunistic pathogenic species. At the family level, *Lachnospiraceae*, *Fusobacteriaceae*, *Prevotellaceae*, and *Bacteroidaceae* were the most dominant, indicating their critical role in the gut ecosystem of dogs (Figure 1).

Protein type did not affect the diversity of microbial species but did not preclude the possibility of differences in specific microbial populations or functional capacities. The stability of alpha diversity across different conditions within this study (Figure 2) suggested that the HFM did not have a negative impact on the gut microbiome (Suchodolski 2011) [49]. In a previous study, Balouei et al. [25] examined the effects of substituting 7% of PM with HFM on the performance and fecal microbiota of six adult female English Setter dogs. Over a 45-day period, no significant differences were found for alpha diversity between diets and Diets × Times interaction, although the analysis revealed higher evenness in the HFM group. Similar findings to the current study were reported by Pinto et al. (2022) [50] and Hsu et al. (2024) [51].

Indeed, the structure of microbial community, measured as beta diversity (Figure 3) was affected by HFM. In agreement with the previous study by Balouei et al. [25], which also found differences in beta diversity following the substitution of PM with HFM. The effect of including hydrolyzed protein on beta diversity was reported Pinto et al. (2022), Hsu et al. (2024), Martínez-López et al. (2021) [50,51,52]. To assess the taxa affected by dietary treatments and sex, a LEfSe analysis was performed (Figure 4).

Different amino acids composition between these two raw materials [25] can affect sequence, type, charge, and dimensional arrangement of the proteins that determine cleavage sites. Moreover, feathers were treated with pressure and steam, a process that is known to decrease the digestibility of protein and to modify several amino acids, with the production of new disulphide linkages, amid bonds, that reduces the enzymatic hydrolysis in the gastrointestinal tract [53]. Treatment applied to HFM could negatively affect the availability of sulphur amino acids, particularly cysteine, that is sensitive to the technological treatments (Moritz and Latshaw 2001) [54]. These factors can affect the extent of undigested protein and the type of peptides into the bowel and, in turn, influence the microbiota composition, as reported in the systematic of Wu et al. [55] that included six mouse studies, seven pig studies, 15 rat studies, and one in vitro study. In a study by Do et al. [56], a comparison of human-grade pet foods with extruded diets revealed that human-grade diets improved digestibility, reduced fecal output, and significantly altered fecal microbiota composition. Notably, dogs fed human-grade beef exhibited a higher relative abundance of *Bacteroidetes* and a lower abundance of *Firmicutes* compared to those receiving fresh or human-grade chicken diets. While the phyla *Actinobacteria*, *Fusobacteria*, *Proteobacteria*, and *Spirochaetes* remained stable, nearly 20 bacterial genera showed changes in RA. In comparison between natural and commercial diets, Kim et al. [57] observed significant differences in fecal microbiota diversity and composition. Dogs on the natural diet exhibited higher alpha diversity and distinct beta diversity, with increased levels of *Clostridium perfringens* and *Fusobacterium varium*. In dogs, a meat-based diet led to reduced fecal weight, improved protein and energy digestibility, and notable changes in fecal microbiota in comparison to a kibble diet. Specifically, *Bacteroides, Prevotella, Peptostreptococcus,* and *Faecalibacterium* decreased, whereas *Fusobacterium*, *Lactobacillus*, and *Clostridium* increased [58]. Ref. [59] reported that a raw meat diet caused an increase of RA of *Fusobacterium* and *Bacteroides*, *Megamonas*, *Lactococcus,* and *Escherichia,* and a reduction of *Lactobacillus, Prevotella,* and *Paralactobacillus*. In a meta-analysis of 16 studies involving 314 dogs [5], dietary protein contents were found to have a significant impact on specific microbial taxa than on overall community diversity. Higher protein intake was associated with increased relative abundances of *Prevotellaceae* Ga6A1 and *Enterococcus*. In research, where chicken meals were partly substituted with chicken liver and hydrolysate, the RA of *Clostridiales*, *Fusobacteriaceae,* and *Bacteroides* decreased, whilst *Lachnospiraceae* increased (Hsu et al., 2024) [51]. Conversely, Pinto et al. (2022) [50] did not report significant variations of fecal microbiota in dogs fed diets either with hydrolyzed chicken liver or with poultry byproduct meal and bovine meat and bone meal-based diet. It must emphasized that the increase of taxa of *Fusobacteriaceae*, a marker of gut health (Pilla and Suchodolski (2021) [60], could suggest no negative effect HFM in the diet of dogs, *Enterobacteriaceae* were higher in the TRT diet, but in the study of Hankel et al. (2020) [61] and Balouei et al. (2024) [25] no significant increase of this family was observed. *Enterobacteriaceae*, members of the *Proteobacteria* phylum, are not only a family of pathogenic bacteria, but are involved in the degradation of carbohydrate and protein and in the maintenance of oxygen homeostasis in the gut of healthy dogs (Moon et al., 2018) [3].

The variations of microbiota in relation to protein source and content seem not to obey to a strict rule, and conflicting results were reported among different studies, depending on the experimental setting, as time of sampling, number of animals, age, sex and diet composition, and in general to environmental factors, including ambient microbiota, that are not easy to compare.

The effects of dietary inclusion of HFM on the fecal microbiome in dogs were also investigated by Hankel et al. (2020) [61], and the authors did not find significant variations in diversity or RA of taxa, likely due to the lower amount of feather meals (2.7%) used in comparison to the present study. Furthermore, present results do not agree with those obtained in a previous study [25], where the TRT diet caused a reduction of RA of *Ruminococcus gnavus*, *Bacteroides coprophilus, Colinsella stercoris,* and *Streptococcus* and an increase *Bacteroides uniformis*. Only for *Peptostreptococcaceae* the increase in the TRT group in comparison to CTR group agree with previous observations. The difference between the two studies mainly lies in sex of the dogs, since the previous trial was conducted only with females and in the present trial dogs were males and females. If this was the reason of the differences is hard to state, but, interestingly, the beta diversity of microbiota was significantly different between sexes (Figure 3). There is limited literature related to the effect of sex on fecal microbiota in dogs. Scarsella et al. [24] found significant differences between male, female, and castrated dogs, while Jah et al. [62] did not observe variations in diversity between genders in a population of household pets in the United States. However, the authors conducted the study by collecting fecal samples from dogs living in very different conditions, such as diet and living environment, which could have masked the relationship between microbiota and sex. Similarly, Pereira et al. [63] reported no effect of sex on fecal microbiota in growing puppies. The role of sex in shaping gut microbial populations has been investigated more extensively in humans. D’Afflitto et al. [64], in a systematic review, reported an association between sex hormones and microbiota composition or diversity, a relationship that was also detected in mice [65]. In particular, the concentration of estrogens in women was associated with enhancement of *Bacteroidetes* and decrease of *Firmicutes*, *Ruminococcaceae,* and increase diversity. In men, testosterone was positively associated to *Ruminococcus*, *Acinetobacter,* and diversity. Differences in gut dimension between female and male dogs in a factor that could affect the transit time of food, thus interacting with microbiome. This finding suggests that sex-specific factors may influence gut microbiota composition, potentially impacting how male and female dogs respond to dietary interventions. Specific taxa differed between the groups, with notable variations in bacterial abundance. The effect of sex on gut microbiome in dogs deserves specific studies to assess which can be the anatomical and endocrine factors which can shape microbial population.

## 5. Conclusions

This study confirms that it is possible to partly substitute PM with HMF part of the dietary protein in the diet of dogs without negatively affecting the dogs’ health and implies that HFM can be a viable alternative protein source, potentially offering benefits such as cost-effectiveness or sustainability compared to traditional protein sources. More studies are required in the future to investigate the effects of the type of processing of HFM on microbiome, also with dose response evaluation. These studies must be extended to other protein sources to evaluate the effect of different industrial processes on microbial compositions.

## Figures and Tables

**Table 1:** Chemical composition (%, as-fed) and content of metabolizable energy (ME) of the poultry hydrolyzed feather meal and of the experimental diets.

		Diets ^‡^	
	Hydrolyzed Feather Meal	PM ^§^	HFM ^¶^
DM	94.41	90.84	91.92
CP	82.64	19.07	19.20
Fat	7.07	15.24	15.00
CF	0.91	2.10	2.00
Ash	1.83	5.45	5.01
Starch	5.63	40.59	40.80
OM	92.63	85.39	86.91
TDF	2.38	7.51	6.97
IDF	2.03	6.14	5.98
SDF	0.35	1.37	1.00
ME, kcal/kg	3562	3677	3682

DM: Dry Matter; CP: Crude protein; CF: Crude Fiber, OM: Organic Matter; TDF: Total Dietary Fiber; IDF: Insoluble Dietary Fiber, SDF: Soluble Dietary Fiber. ME: Metabolizable Energy. ^‡^ Extruded commercial dry diets. Ingredients: Cereals pregelatinized (rice 30%, starch digestibility 95%; malted cereals 0.3%), processed animal proteins of poultry origin 16%, Oils and fats, Vegetables, Carob extract (roasted), Fish and fish byproducts, Extruded flax, Vitamins and Minerals, *Saccharomyces cerevisiae*, Algae, Yucca shidigera; Extracts of *Andrographis paniculata*, *Boerhavia diffusa*, *Physilantus amarus*, *Solanum nigrum*, Silymarin. ^§^ PM diet: diet with poultry meal (16%). ^¶^ HFM diet: diet with poultry meal (9%) and hydrolyzed feather meal (7%).

**Table 2:** Amino acid composition of the experimental diets (%, as-fed).

	Diets	
	PM ^‡^	HFM ^§^
Essential Amino Acid		
Arginine	1.62	1.67
Histidine	0.37	0.36
Isoleucine	0.90	0.91
Leucine	1.49	1.50
Lysine	0.80	0.81
Methionine	0.40	0.44
Phenylalanine	1.01	1.03
Tryptophan	0.27	0.27
Threonine	0.72	0.73
Valine	1.23	1.26
Non Essential amino acid		
Serine	2.13	2.20
Proline	1.65	1.72
Alanine	0.65	0.68
Glycine	1.63	1.66
Aspartic Acid + Asparagine	1.23	1.25
Hydroxyproline	0.51	0.53
Glutamic Acid + Glutamine	1.82	1.91
Hydroxylysine	0.45	0.46
Tyrosine	0.57	0.58
Cysteine	0.41	0.42

^‡^ PM diet: diet with poultry meal (16%). ^§^ HFM diet: diet with poultry meal (9%) and hydrolyzed feather meal (7%).

**Table 3:** Mean values and standard deviation of body weight (BW), body condition score (BCS), muscle condition score (MCS), and fecal consistency score (FCS) of the dogs fed control diet (CTR) and experimental diet (TRT) at time of sampling 0, 3, 7, 15, and 45 days.

	BW		BCS		MCS		FCS	
Effects	Mean	SD	Mean	SD	Mean	SD	Mean	SD
Diet								
CTR	19.5	4.1	5.2	0.8	1.1	0.2	2.5	0.2
TRT	19.6	3.0	5.0	0.0	1.0	0.0	2.3	0.3
Sex								
Male	16.6 ^B^	1.1	5.0	0.0	1.0	0.0	2.4	0.2
Female	22.6 ^A^	2.5	5.2	0.8	1.1	0.2	2.4	0.3
Diet × Time of sampling								
CTR_T0	19.3	4.6	5.0	0.8	1.0	0.0	2.4 ^a^	0.4
CTR_T3	19.4	4.6	5.0	0.8	1.0	0.0	2.5 ^a^	0.2
CTR_T7	19.6	4.7	5.5	1.0	1.0	0.0	2.5 ^a^	0.2
CTR_T15	19.8	4.7	5.5	1.0	1.0	0.0	2.5 ^a^	0.3
CTR_T45	19.6	4.6	5.0	0.8	1.3	0.5	2.4 ^a^	0.2
TRT_T0	19.4	3.4	5.0	0.0	1.0	0.0	2.2 ^b^	0.3
TRT_T3	19.5	3.3	5.0	0.0	1.0	0.0	2.4 ^a^	0.2
TRT_T7	19.6	3.5	5.0	0.0	1.0	0.0	2.2 ^b^	0.3
TRT_T15	19.9	3.5	5.0	0.0	1.0	0.0	2.2 ^b^	0.3
TRT_T45	19.7	3.4	5.0	0.0	1.0	0.0	2.4 ^a^	0.2

SD: Standard Deviation. Diet: CTR group, dogs fed with diet with poultry meal); TRT group, dogs fed with diet with hydrolyzed feather meal). Sampling times: T0, T3, T7, T15, and T45 correspond to the samples collected at the beginning of the study and after 3, 7, 15, and 45 days. Within a column, different upper-case letters denote mean significantly different for *p* < 0.01, and different lower-case letters denote mean significantly different for *p* < 0.05.

## Data Availability

The raw sequence data obtained was deposited in the NCBI Sequence Read Archive under the accession number PRJNA1079213.

## Supplementary Materials

The following supporting information can be downloaded at: https://www.mdpi.com/article/10.3390/microorganisms13010121/s1, Figure S1: Rarefaction curve of the sequence reads in dogs fed control diet (CTR) and experimental diet (TRT). CTR group, dogs fed with PM diet (diet with poultry meal); TRT group, dogs fed with HFM diet (diet with hydrolyzed feather meal); Figure S2: Rarefaction curve of the sequence reads in dogs fed control diet (CTR) and experimental diet (HFM) during the study.
