# Supplementary material for: Nutritional and Microbiome Effects of a Partial Substitution of Poultry Meat with Hydrolyzed Feather Meal in Dog Diets
Source: Microorganisms. 2025 Jan 9;13(1):121. doi: 10.3390/microorganisms13010121 (PMC11767478; doi:10.3390/microorganisms13010121)
Supplement: Supplementary file 1 [file microorganisms-13-00121-s001.zip › microorganisms-3314650-supplementary.pdf]

Supplementary Figure S1. Rarefaction curve of the sequence reads in dogs fed control diet (CTR) and experimental diet (HFM).

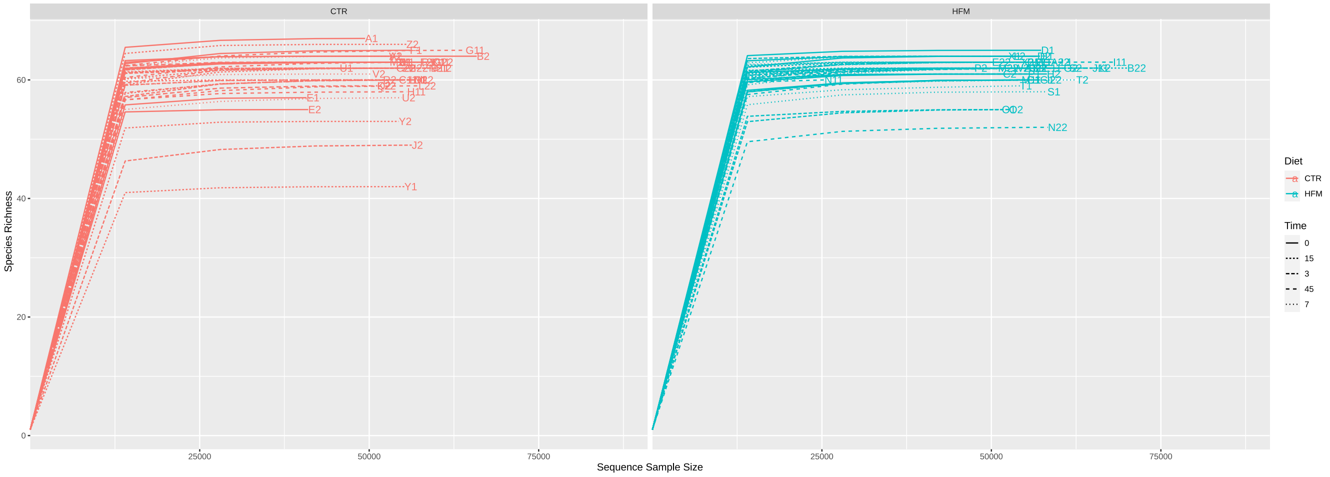

CTR group, dogs fed with PM diet (diet with poultry meal); TRT group, dogs fed with HFM diet (diet with hydrolyzed feather meal).

Supplementary Figure S2. Rarefaction curve of the sequence reads in dogs fed control diet (CTR) and experimental diet (HFM) during the study.

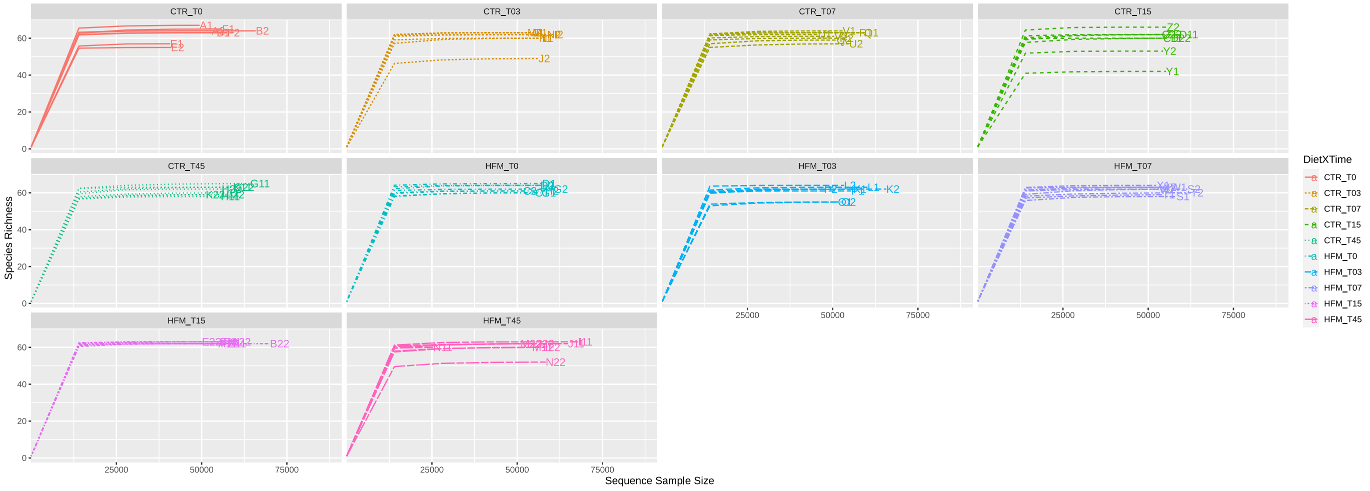

CTR group, dogs fed with PM diet (diet with poultry meal); TRT group, dogs fed with HFM diet (diet with hydrolyzed feather meal).

Times of sampling: T0, T3, T7, T15 and T45 denote samples collected at the beginning of the study and after 3, 7, 15 and 45 days.
